# Relationship between anti-dsDNA, anti-nucleosome and anti-alpha-actinin antibodies and markers of renal disease in patients with lupus nephritis: a prospective longitudinal study

**DOI:** 10.1186/ar2831

**Published:** 2009-10-14

**Authors:** Jessica J Manson, Alexander Ma, Pauline Rogers, Lesley J Mason, Jo H Berden, Johan van der Vlag, David P D'Cruz, David A Isenberg, Anisur Rahman

**Affiliations:** 1 Centre for Rheumatology Research, University College London, Windeyer Institute, 46 Cleveland Street, London W1T 4JF, UK; 2 Joint University College London Hospital/University College London and Royal Free Biomedical Research Unit, Research and Development (1st Floor Maple House), Rosenheim Wing, 25 Grafton Way, London WC1E 6DB, UK; 3 Nephrology Research Laboratory, Nijmegen Centre for Molecular Life Sciences, Department of Nephrology, Radboud University Nijmegen Medical Centre, PO Box 9101, 6500 HB Nijmegen, The Netherlands; 4 Lupus Research Unit, The Rayne Institute, St Thomas' Hospital, Lambeth Palace Road, London SE1 7EH, UK

## Abstract

**Introduction:**

Glomerulonephritis is a major cause of morbidity and mortality in patients with systemic lupus erythematosus (SLE). Deposition of autoantibodies in the glomeruli plays a key role in the development of lupus nephritis (LN). Different groups have proposed that either anti-nucleosome antibodies or antibodies that bind the intrinsic renal antigen, α-actinin, are central to the pathogenesis of LN. These theories have been based mainly on cross-sectional studies in patients and on experiments in animal models. No previous longitudinal studies have compared the relationships between levels of these antibodies and markers of renal function. We assessed how well anti-α-actinin, anti-nucleosome and anti-double-stranded DNA (anti-dsDNA) antibodies reflected renal outcome measures in patients with new-onset LN followed for up to 2 years.

**Methods:**

Renal disease activity was monitored by measuring urine protein/creatinine ratio (PCR), serum albumin and a composite outcome of renal remission. At each time point, anti-nucleosome and anti-α-actinin antibodies were measured by enzyme-linked immunosorbent assay. High-avidity anti-dsDNA antibodies were measured using the Farrzyme assay. We analysed relationships between levels of the three antibodies and between antibody levels and renal outcome measures over time.

**Results:**

Levels of anti-nucleosome and anti-dsDNA were positively correlated with each other (*r *= 0.6, *P *= 0.0001) but neither correlated with anti-α-actinin level. At baseline, mean anti-nucleosome levels were higher in patients with LN than in healthy controls (0.32 versus 0.01, *P *< 0.001). The same was true for anti-dsDNA antibodies (0.50 versus 0.07, *P *< 0.001) but not for anti-α-actinin (0.33 versus 0.29). Over the follow-up period, anti-nucleosome and anti-dsDNA levels associated positively with urine PCR (*P *= 0.041 and 0.051, respectively) and negatively with serum albumin (*P *= 0.027 and 0.032, respectively). Both anti-nucleosome and anti-dsDNA levels were significantly lower during renal remission than when renal disease was active (*P *= 0.002 and 0.003, respectively). However, there was no relationship between anti-α-actinin levels and urine PCR, serum albumin or remission status.

**Conclusions:**

This prospective longitudinal clinical study is the first to compare levels of anti-nucleosome, anti-dsDNA and anti-α-actinin antibodies in the same patients with SLE. Our results support the concept that, in the majority of patients, anti-nucleosome antibodies play a major role in pathogenesis of LN, in contrast to anti-α-actinin antibodies.

## Introduction

Lupus nephritis (LN) occurs in 40% to 60% of patients with systemic lupus erythematosus (SLE) [1]. Koffler and colleagues [2] first demonstrated deposition of autoantibodies in LN renal tissue. A range of evidence from clinical [3], renal biopsy [4] and animal [5-7] studies suggested that anti-double-stranded DNA (anti-dsDNA) antibodies were the main autoantibodies involved in the pathogenesis of LN. It has been argued that high-avidity anti-dsDNA antibodies are particularly linked to pathogenicity, and some laboratory tests have been developed specifically to test for these high-avidity antibodies [8]. However, there are clearly some patients with persistently high anti-dsDNA levels who never develop LN [9] and there is no simple relationship between the ability of passively transferred monoclonal antibodies to bind dsDNA and the ability of the same antibodies to cause glomerulonephritis [5-7].

In some cases, modification of antibodies by mutagenesis increased binding to dsDNA but reduced pathogenicity [7]. In other cases, pathogenic monoclonal antibodies were found not to bind dsDNA at all after rigorous purification and were actually anti-nucleosome antibodies [10,11]. Furthermore, when a rat kidney perfusion system was used, glomerular binding of monoclonal antibodies was shown to require the presence of nucleosomes [12]. It has therefore been argued that binding to nucleosomes is a major determinant of pathogenicity of autoantibodies in LN [13,14].

An alternative theory holds that direct cross-reaction of anti-dsDNA with intraglomerular antigens is key [13,15]. Although cross-reactivity with a number of proteins (including laminin and type IV collagen) has been postulated (reviewed in [13]), the importance of anti-α-actinin antibodies has been particularly stressed in recent years. This emphasis on the possible pathogenic role of anti-α-actinin antibodies has arisen as a result of studies in murine models [6,16] and clinical studies [17-19], although anti-α-actinin antibodies could not be eluted from glomerular deposits in mice with LN [20]. However, no previous study has compared anti-nucleosome and anti-α-actinin antibody levels in the same patients.

In this study, we identified 16 patients with new-onset LN and followed them prospectively for up to 2 years. We tested their blood for both anti-nucleosome and anti-α-actinin antibodies, allowing (for the first time) direct comparison of both of these important specificities in the same patients with LN. Furthermore, we examined the associations between levels of both anti-nucleosome and anti-α-actinin antibodies, levels of high-avidity anti-dsDNA antibodies and markers of renal disease, including an assessment of whether the patients entered renal remission.

## Materials and methods

Sixteen patients with new-onset biopsy-proven LN were recruited prospectively from the Lupus Clinics at University College London and St Thomas' hospitals (London). All of the patients fulfilled the American College of Rheumatology revised criteria for SLE [21,22]. Blood samples were taken at the time of recruitment and then at routine follow-up appointments and were spun at 500 *g *for 10 minutes to produce serum, which was stored at -20°C. To establish normal values for the enzyme-linked immunosorbent assays (ELISAs) used, 30 healthy volunteer donors with age and gender distributions similar to those of the controls were recruited from staff at University College London Hospital and University College London. Serum was collected and stored as above. Patients and healthy controls gave informed consent. This study received approval from the Thames Valley Multi-Centre Research Ethics Committee (reference number 04/MRE12/58) and was passed by the Joint University College London/University College London Hospitals Committee on the Ethics of Human Research and the St Thomas' Hospital Research Ethics Committee.

### Renal outcome measures

For each patient at each time point, urine was tested for protein/creatinine ratio (PCR), and serum was tested for albumin and creatinine in the routine clinical laboratory. Statistical analysis was carried out on three outcome measures: absolute values of urine PCR and serum albumin and a composite score for renal disease activity. This composite score was defined using measurements of urine PCR and serum albumin and creatinine. Complete renal remission was defined as follows: PCR of not more than 30 mg/mmol, normal serum albumin and normal serum creatinine. Partial remission was defined as follows: decrease in urine PCR by at least 50%, serum albumin of at least 30 g/L, and either normal serum creatinine if the baseline creatinine was less than 260 μmol/L or a 50% decrease in creatinine if the baseline value was at least 260 μmol/L. Patients who did not fulfil these criteria for either complete or partial remission were considered to have active LN.

### Human IgG anti-nucleosome enzyme-linked immunosorbent assay

Serum samples were diluted 1:800 in phosphate-buffered saline/0.05% Tween 20 (PBS-T) and tested in duplicate for binding to nucleosomes prepared from Jurkat cells. The methods for obtaining nucleosomes and carrying out the ELISA have been described previously [9,23]. Monoclonal human IgG antibodies with well-defined anti-nucleosome-binding properties [23] were used as positive and negative controls. To standardise results between ELISA plates, readings were taken when the positive control had reached an optical density (OD) of approximately 1.2.

### Human IgG anti-α-actinin enzyme-linked immunosorbent assay

Serum samples were diluted 1:100 in PBS-T and tested in an anti-α-actinin ELISA. The ELISA method was as described previously [17]. Again, the positive and negative controls were human monoclonal antibodies produced in our laboratory with known anti-α-actinin-binding properties [23]. Plates were read when the OD of the positive control reached 1.2.

### Detection of high-avidity human IgG anti-double-stranded DNA antibodies

High-avidity anti-dsDNA antibody titre was measured using the Farrzyme assay (The Binding Site, Birmingham, UK) in accordance with the instructions of the manufacturer [8].

### Expression of results of enzyme-linked immunosorbent assay tests

ODs from all of the assays were converted to absorbance ratios (ARs) to standardise the data and minimise interassay variation. The mean OD for each sample was calculated from the duplicates. The mean OD was then divided by the standard positive control on that assay plate to give the AR.

### Statistical analysis

The data were analysed in GraphPad Prism (GraphPad Software Inc., San Diego, CA, USA) and using the 'xt' commands for longitudinal data in Stata 9.2 (StataCorp LP, College Station, TX, USA). The aim of the analysis was to assess which antibody level (high-avidity anti-dsDNA, anti-nucleosome or anti-α-actinin) best reflected the renal outcome measures, PCR, albumin and remission status. First, the continuous laboratory variables (all three antibodies, albumin and PCR) were tested for normality and, if necessary, were transformed using log transformation. PCR measurements and ARs for anti-nucleosome and high-avidity anti-dsDNA antibodies were so transformed, and consequently changes in these measurements are presented as percentages rather than as absolute values. Mean ARs for the baseline binding data in the patient and control groups were compared using the Student *t *test using the Satterthwaite approximation for unequal variances where appropriate. Possible correlations between the results of the three antibody assays were investigated by calculating the Pearson correlation coefficient, using log-transformed data where appropriate. For the two continuous outcome variables (albumin and PCR), each explanatory variable was analysed one at a time and significant variables then were included in a multivariable sensitivity analysis. Maximum likelihood random effects models were used to fit linear regression models with random intercepts. The residuals from all models were checked for normality using a normal plot. The relationship between remission status and the three laboratory explanatory variables was investigated using the regression specification for a one-way analysis of variance. Anti-nucleosome level, anti-α-actinin level and high-avidity anti-dsDNA level were each considered in turn as the outcome with remission as the explanatory variable. The Wald test was used to test contrasts between the coefficients for the different levels of renal remission (active disease, partial remission and complete remission). Due to the size of the dataset, there was insufficient statistical power to carry out multivariable analysis, and significant results from these analyses should be treated with caution.

## Results

### Characteristics of patients and control subjects

Sixteen patients were enrolled, and baseline details for all patients are given in Table 1. There were 15 females and one male. The mean age was 33.4 years (standard deviation [SD] 10.9, range 18 to 56). Three of the patients were Black, six were South Asian and seven were White. The mean age of the 30 healthy control subjects was 39 years (SD 11.5, range 24 to 64). There were 24 females and six males. Five were Black, four were South Asian and 21 were White. Renal biopsies were classified in accordance with the World Health Organization criteria [24]. Two patients had class II disease, seven had class III or IV, three had pure class V and four had class V with III or IV. At baseline, the mean urine PCR was 285 mg/mmol (range 34 to 1,017), and the mean serum albumin was 31 g/L (range 17 to 44). Medications at time of enrolment are shown in Table 1. All patients but one were taking oral prednisolone (daily dose range 5 to 60 mg), and 12 were also treated with immunosuppressants (mycophenolate, cyclophosphamide or azathioprine). Patient LN11 differed from all of the others in several important respects. He was the only man, the only patient with increased serum creatinine at baseline (156 μmol/L) and the only patient to require renal dialysis. Data from this patient are nevertheless included in all of the analyses below except where stated specifically. Anti-nucleosome and high-avidity anti-dsDNA but not anti-α-actinin level were higher in patients with LN than in healthy controls.

**Table 1:** Baseline patient data

Patient ID	Gender	Ethnicity	Biopsy result	Urine PCR, mg/mmol	Serum albumin, g/L	Treatment, daily dose in mg
LN1	Female	Asian	III	58	38	P (20), H (400)
LN2	Female	White	IV, V	457	30	P (20), MP^a^
LN3	Female	Black	V	123	28	P (30), H (400)
LN4	Female	White	IV	533	21	P (10), H (400), MMF (1,500)
LN5	Female	White	V	569	23	P (10), H (400)
LN6	Female	Black	III	202	30	P (5), C^a^
LN7	Female	Asian	III	96	20	P (15), A (100)
LN8	Female	Asian	III/V	208	36	P (12.5), H (200)
LN9	Female	Asian	III	34	28	P (60), C^a^
LN10	Female	Asian	IV	66	44	P (10), MMF (2,000)
LN11	Male	Asian	IV	1,017	17	P (20), M (1,000), C^a^
LN12	Female	White	II	312	34	H (400)
LN13	Female	White	II	127	33	H (400), MP^a^
LN14	Female	White	V	366	34	P (20), A (100)
LN15	Female	White	III/V	88	43	P (5), A (100)
LN16	Female	Black	IV/V	297	37	P (20)

^a^MP and C signify a recent intravenous pulse of methylprednisolone or cyclophosphamide, respectively; thus, there is no value for daily dose. Values for daily dose of each drug refer to the dose being taken at the time of entry into the study. A, azathioprine; H, hydroxychloroquine; LN, lupus nephritis; MMF, mycophenolate mofetil; P, prednisolone; PCR, protein/creatinine ratio.

**Figure 1:**
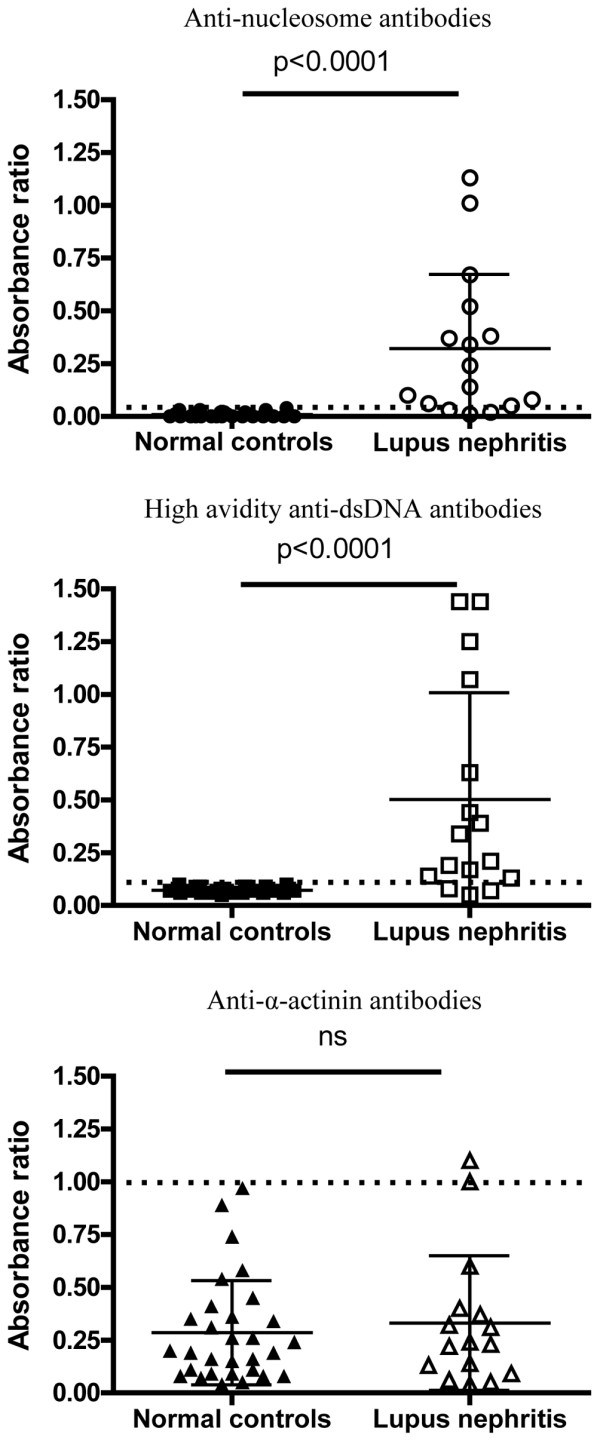
Baseline binding data for binding to nucleosomes, double-stranded DNA and α-actinin. On each graph, absorbance ratios of the 30 normal controls and the 16 patients with lupus nephritis are plotted. The mean and standard deviation are also plotted. The groups were compared using the Student *t *test. The dotted black line shows the upper limit of normal for each assay. anti-dsDNA, anti-double-stranded DNA; ns, not significant.

Baseline binding data from all patients were compared with the results from 30 normal controls (Figure 1). The mean (SD) ARs for anti-nucleosome antibodies were significantly higher for LN patients than controls (means were calculated on raw data, but log-transformed data were analysed where appropriate) (0.32 [0.35] versus 0.01 [0.01], *P *< 0.0001) as were the high-avidity anti-dsDNA AR (0.50 [0.50] versus 0.07 [0.01], *P *< 0.0001) but not anti-α-actinin (0.33 [0.32] versus 0.29 [0.25]). For each assay, the upper limit of normal was taken as the mean plus three SDs of the results from the 30 normal controls. At baseline, 13 out of 16 patients were above this cutoff in the anti-nucleosome and high-avidity anti-dsDNA assays, whereas only two had anti-α-actinin levels that were above the upper limit of normal.

### Changes in antibody levels over the follow-up period

Follow-up dates were determined by the need of the patient to attend the clinic. The mean number of weeks over which the patients were followed was 37.3 (range 10 to 85). The mean interval between test points was 9.3 weeks (range 2 to 26). All patients had samples collected on at least three occasions, with a median number of time points per patient of 4.5 (range 3 to 8). On average, all three assays demonstrated significant downward linear trends over time. Anti-nucleosome antibodies decreased by 1.6% per week, and high-avidity anti-dsDNA antibodies decreased by 0.8% per week. Anti-α-actinin antibodies decreased by 0.0015 per week, which (given the mean baseline AR for anti-α-actinin antibodies of 0.25) is the equivalent of a 0.6% decrease per week. In general, if anti-nucleosome antibodies were high for any patient at any time point, so were high-avidity anti-dsDNA antibodies. By contrast, high anti-α-actinin antibodies (taken as an AR of at least 0.5) were detected in only three patients (LN3, LN9 and LN15) and were seen in combination with low AR for the other two assays. Six patients had low titres (AR <0.25) of anti-nucleosome and high-avidity anti-dsDNA antibodies throughout the study period.

**Figure 2:**
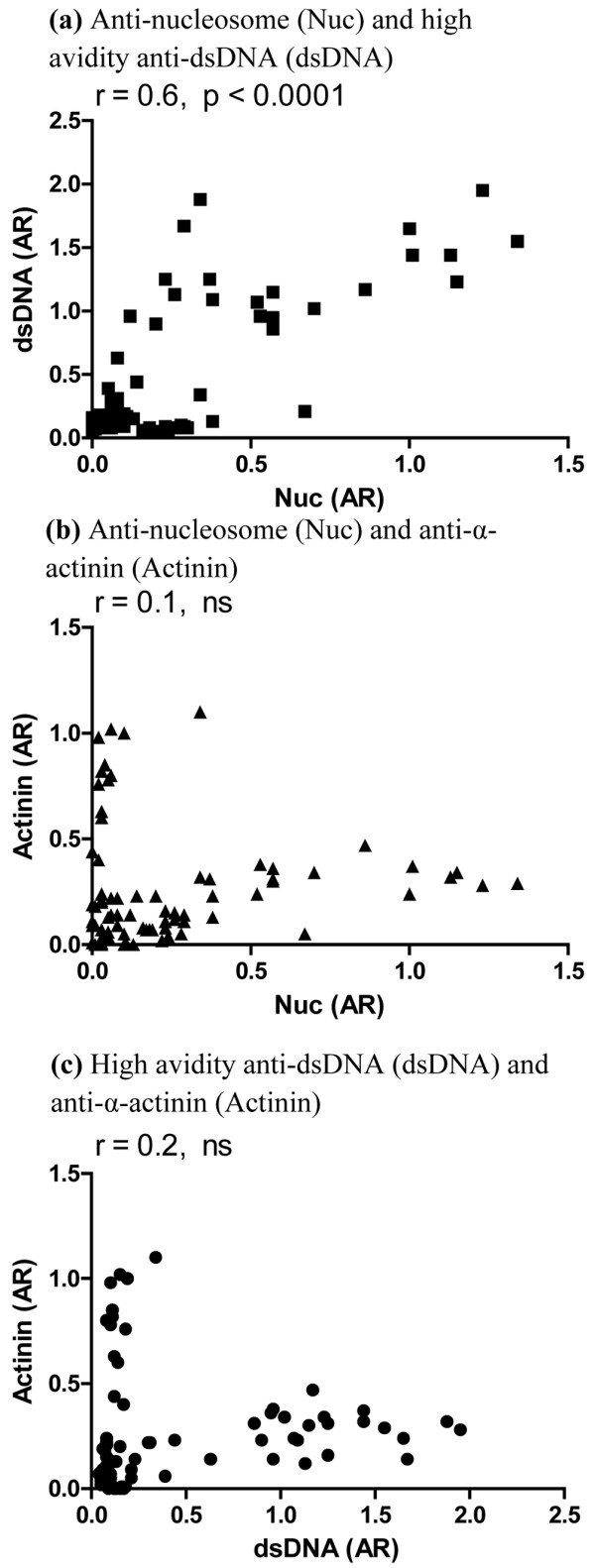
Correlation between binding to (a) nucleosomes and double-stranded DNA (dsDNA), (b) nucleosomes and α-actinin and (c) dsDNA and α-actinin. Absorbance ratios (ARs) for each assay are plotted against each other. The Pearson correlation coefficient is given for each association (*r*). ns, not significant.

Consistent with these findings, analysis of the whole dataset showed significant positive correlation between anti-nucleosome and high-avidity anti-dsDNA levels (*r *= 0.6, *P *= 0.0001) but no correlation between anti-nucleosome and anti-α-actinin or high-avidity anti-dsDNA and anti-α-actinin (Figure 2). Anti-nucleosome and high-avidity anti-dsDNA levels associate positively with urine PCR and negatively with serum albumin over time. Analysis of the relationship between high-avidity anti-dsDNA antibody titre and urine PCR revealed a significant positive linear trend (*P *= 0.041). The relationship between anti-nucleosome antibodies and PCR reached borderline statistical significance (*P *= 0.051). When we repeated the analysis excluding patient LN11, both associations were statistically significant: *P *= 0.021 for anti-nucleosome and *P *= 0.043 for high-avidity anti-dsDNA. On average, urine PCR increased by 18% for every twofold increase in anti-nucleosome level and by 27% for every twofold increase in anti-dsDNA level.

There were significant negative linear trends between albumin levels and anti-nucleosome (*P *= 0.027) and high-avidity anti-dsDNA (*P *= 0.032) antibody titre. Repeating the analysis after exclusion of patient LN11 confirmed the analysis and showed stronger levels of statistical significance (*P *= 0.001 and 0.011, respectively). On average, albumin decreased by 1.16 g/L for every twofold increase in anti-nucleosome titre and by 1.59 g/L for every twofold increase in high-avidity anti-dsDNA antibody titre. There was no relationship between anti-α-actinin levels and either urine PCR (*P *= 0.401) or serum albumin (*P *= 0.332).

### Remission status

Analysis of the three-level remission score (active disease, partial remission and complete remission) demonstrated significant differences between the groups in all three antibody levels (anti-nucleosome, *P *= 0.001; high-avidity anti-dsDNA, *P *= 0.0063; and anti-α-actinin, *P *= 0.0368). Application of the Wald test (which assesses whether there is a true difference between groups) suggested that there was no real difference in the levels between partial and complete remission. The partial and complete remission categories were combined such that there were only two possible renal activity levels for each patient at each time point (active or remission), and the data were then re-analysed. The association between activity status and anti-nucleosome or high-avidity anti-dsDNA antibody titre was maintained (*P *= 0.002 and 0.003, respectively), but there was no observed difference in the levels of anti-α-actinin antibodies between active disease and remission. On average, anti-nucleosome levels were 53.8% lower and high-avidity anti-dsDNA levels were 34.5% lower when patients were in remission than when they had active disease.

### Longitudinal studies in individual patients

**Figure 3:**
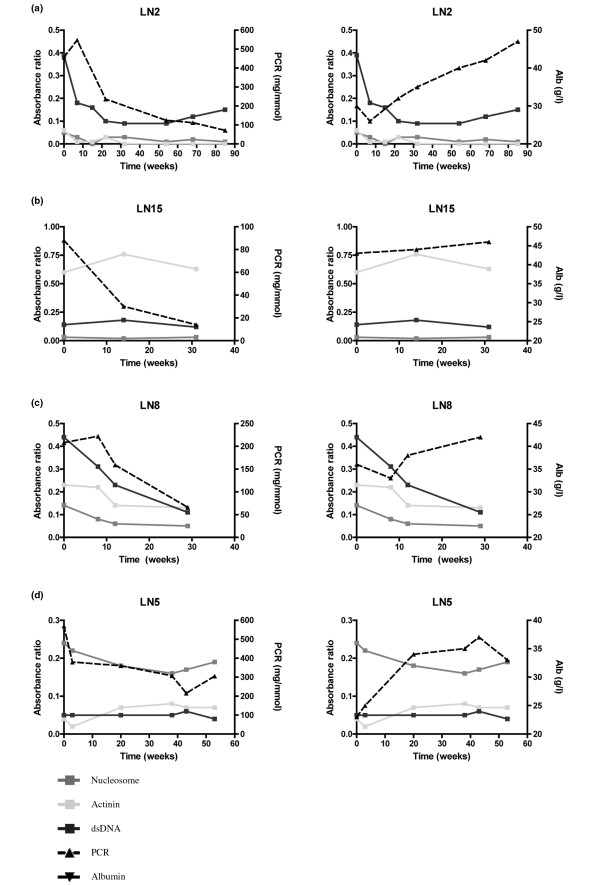
Patterns of relationship between antibody titre and outcome in individual patients. Graphic display of enzyme-linked immunosorbent assay data (anti-nucleosome, anti-α-actinin and high-avidity anti-double-stranded DNA [Farrzyme]) with urine protein/creatinine ratio (PCR) or serum albumin (Alb). dsDNA, double-stranded DNA; LN, lupus nephritis.(a) Patient LN2 - all three antibody levels remained low. (b) Patient LN15 - anti-α-actinin high, others low. (c) Patient LN8 - anti-dsDNA levels mirror changes in albumin and PCR. (d) Patient LN5 - anti-nucleosome levels mirror changes in albumin and PCR.

Several different patterns were discernible. In three patients (LN2, LN12 and LN14), levels of all three antibodies remained low from baseline throughout the follow-up period and were not closely related to outcome measures. An example of this pattern is illustrated in Figure 3a. In three other patients (LN3, LN9 and LN15), anti-α-actinin levels remained high throughout the observation period whereas levels of the other two autoantibodies remained low. However, there was no clear relationship between anti-α-actinin levels and serum albumin or urine PCR in these patients and no particular clinical or demographic feature distinguished them from the other 13 patients. An example of this pattern is illustrated in Figure 3b. In the remaining cases, anti-nucleosome or high-avidity anti-dsDNA antibodies or both were increased during the period of observation. In some patients, the decrease in anti-dsDNA antibodies (patient LN8, Figure 3c) or anti-nucleosome antibodies (patient LN5, Figure 3d) mirrored changes in serum albumin and urine PCR.

## Discussion

This is the first clinical study to achieve a direct comparison of the associations between anti-nucleosome, high-avidity anti-dsDNA and anti-α-actinin levels and outcomes of renal disease in patients with LN followed longitudinally. The results show that assessment of any renal outcome measure (whether serum albumin, urine PCR or remission) over time favoured the theory that renal disease activity was linked to the presence of anti-nucleosome and anti-dsDNA antibodies and not to anti-α-actinin antibodies.

Nucleosomes released in apoptotic debris, but not cleared efficiently from the circulation [25], are critical immunogenic stimulants for both T cells and B cells in SLE [13,26]. The mechanism by which anti-nucleosome antibodies bind glomeruli has been reviewed extensively elsewhere [13,14]. Recent electron microscopy data from renal biopsies of both human and murine LN confirm that autoantibodies in those tissues co-localise with electron-dense extracellular deposits of chromatin [27,28]. High titres of anti-nucleosome antibodies have been found in up to 87% of patients with SLE [26,29,30], and some studies have noted a particular association with renal disease [31,32]. When highly purified nucleosomes are used as the antigen, the anti-nucleosome assay is very specific for patients with SLE [33]. However, these serological studies either were cross-sectional or did not evaluate quantitative markers of renal impairment [30].

A Dutch study of 52 patients with proliferative LN [34], followed longitudinally for a year as part of a clinical trial, showed good correlation between these two serological measures (*r *= 0.63, *P *< 0.001), as in our study. Data on renal outcomes across the whole patient group were reported in terms of relapse or remission, but data on serum albumin and urine PCR were not given. The investigators did not observe increases in anti-nucleosome or anti-dsDNA antibody titre prior to renal relapse. There was no association between levels of anti-nucleosome antibodies at disease entry and occurrence of relapse or time to remission. Data on individual patients were not given.

Alpha-actinin-4 is an actin-binding protein present in both podocytes and mesangial cells. Two groups showed that the ability of murine monoclonal anti-dsDNA or anti-nucleosome antibodies to cause pathogenicity in mice was related to their ability to cross-react with α-actinin [6,16], and one of the groups then showed that a cross-reactive human anti-dsDNA/anti-α-actinin antibody caused glomerulonephritis in these mice [35]. However, the electron-dense deposits that are the sites of autoantibody deposition in lupus-prone NZB/W F1 mice do not co-localise with α-actinin. Three previous clinical studies have looked at anti-α-actinin levels in patients with SLE [17-19]. One study showed that purified anti-dsDNA antibodies from patients with SLE were more likely to cross-react with α-actinin if the patients had nephritis [17]. Two subsequent studies [18,19] were both cross-sectional. Both groups showed that anti-α-actinin antibodies can occur in patients with diseases other than SLE (for example, rheumatoid arthritis [19] and autoimmune hepatitis [15]) and occur in both patients with LN and patients with lupus but not nephritis. In one study, positivity for anti-α-actinin distinguished patients with nephritis from those without nephritis more clearly than positivity for anti-dsDNA, although only 10 out of 24 patients with nephritis were positive for anti-α-actinin [18]. Neither study looked at anti-nucleosome antibody levels, and neither showed any correlation between levels of anti-α-actinin antibodies and indicators of renal disease such as proteinuria. To our knowledge, ours is the first longitudinal study of anti-α-actinin levels in patients with LN. Although our results do not favour an important role for cross-reactive anti-α-actinin antibodies in most patients with LN, they leave open the possibility that these antibodies may be important in a minority of such patients. Three of our 16 patients had increased levels of anti-α-actinin antibodies but not anti-dsDNA or anti-nucleosome antibodies. This is in contrast to the study of Renaudineau and colleagues [18], who found that 21 out of 22 patients with SLE who had increased anti-α-actinin levels also had increased anti-dsDNA. Fewer than half of these 22 patients had LN.

Although our results are important in shedding more light on the pathogenesis of LN, they do not suggest any changes in clinical practice. Neither anti-nucleosome nor anti-α-actinin tests seem likely to be better predictors of renal outcome than anti-dsDNA ELISA, which is already widely used for monitoring patients with SLE. Improved monitoring of renal disease in patients with LN is more likely to be achieved by combining anti-dsDNA tests with assays more specific for renal dysfunction, such as urinary gelatinase B-associated lipocalin (nGAL), which has been shown to be a marker of renal disease in cross-sectional studies of both adult [36] and paediatric [37] SLE.

## Conclusions

This is the first prospective longitudinal study of patients with new-onset biopsy-proven LN to study antibody levels and renal outcome measures at multiple time points within the first two years after diagnosis. In particular, we measured both anti-nucleosome and anti-α-actinin levels, specificities that have been studied previously in separate groups of patients with LN but never in the same group. The most important conclusion of our study is that anti-nucleosome and high-avidity anti-dsDNA antibodies are much more closely related to renal outcome measures in the majority of these patients than anti-α-actinin levels.

## Abbreviations

anti-dsDNA: anti-double-stranded DNA; AR: absorbance ratio; dsDNA: double-stranded DNA; ELISA: enzyme-linked immunosorbent assay; LN: lupus nephritis; OD: optical density; PBS-T: phosphate-buffered saline/0.05% Tween 20; PCR: protein/creatinine ratio; SD: standard deviation; SLE: systemic lupus erythematosus.

## Competing interests

The authors declare that they have no competing interests.

## Authors' contributions

JJM helped to recruit patients for the study and to obtain samples, to carry out immunoassays and statistical analyses, to conceive and design the study and to write the final manuscript. DPD helped to recruit patients for the study and to obtain samples. AM and LJM helped to carry out immunoassays. PR helped to carry out statistical analyses. JHB and JV originally developed the anti-nucleosome ELISA and advised on immunoassays and design of the study. AR helped to conceive and design the study and to write the final manuscript. DAI helped to conceive and design the study. All authors read and approved the final manuscript.
